# A call for collaboration and consensus on training for endotracheal intubation in the medical intensive care unit

**DOI:** 10.1186/s13054-020-03317-3

**Published:** 2020-10-22

**Authors:** Wade Brown, Lekshmi Santhosh, Anna K. Brady, Joshua L. Denson, Abesh Niroula, Meredith E. Pugh, Wesley H. Self, Aaron M. Joffe, P. O’Neal Maynord, W. Graham Carlos

**Affiliations:** 1 Division of Allergy, Pulmonary, and Critical Care Medicine, Vanderbilt University Medical Center, T-1218 Medical Center North, 1211 Medical Center Drive, Nashville, TN 37232 USA; 2 Division of Pulmonary and Critical Care Medicine, University of California San Francisco, San Francisco, CA USA; 3 Division of Pulmonary and Critical Care Medicine, Oregon Health Science University, Portland, OR USA; 4 Section of Pulmonary, Critical Care, and Environmental Medicine, Tulane University School of Medicine, New Orleans, LA USA; 5 Division of Pulmonary, Allergy, Critical Care, and Sleep Medicine, Emory University School of Medicine, Atlanta, GA USA; 6 Department of Emergency Medicine, Vanderbilt University Medical Center, Nashville, TN USA; 7 Department of Anesthesiology and Pain Medicine, University of Washington School of Medicine, Seattle, WA USA; 8 Division of Pediatric Critical Care Medicine, Monroe Carell Jr. Children’s Hospital at Vanderbilt and Vanderbilt University School of Medicine, Nashville, TN USA; 9 Division of Pulmonary, Critical Care, Sleep and Occupational Medicine, Indiana University School of Medicine, Indianapolis, IN USA

**Keywords:** Intubation, intratracheal, Education, Emergency medicine, Critical care, Anesthesiology, Teaching, Critical illness, Laryngoscopy, Manikins, Learning curve, Education, medical, graduate, Consensus, Guideline

## Abstract

Endotracheal intubation (EI) is a potentially lifesaving but high-risk procedure in critically ill patients. While the ACGME mandates that trainees in pulmonary and critical care medicine (PCCM) achieve competence in this procedure, there is wide variation in EI training across the USA. One study suggests that 40% of the US PCCM trainees feel they would not be proficient in EI upon graduation. This article presents a review of the EI training literature; the recommendations of a national group of PCCM, anesthesiology, emergency medicine, and pediatric experts; and a call for further research, collaboration, and consensus guidelines.

## Main text

Endotracheal intubation (EI) is a potentially lifesaving but high-risk procedure in critically ill patients [1]. Complications occur in more than half of all adult intensive care unit (ICU) endotracheal intubations with severe hypoxemia in 26% and hemodynamic collapse in 25% [2]. At the extreme, cardiac arrest occurs in up to 3% and death in up to 1% of patients [2, 3]. These rates reflect the anatomic, physiologic, and situational complexity of EI in the critically ill patient [4–6]. While the ACGME mandates that trainees in pulmonary and critical care medicine (PCCM) be competent in this procedure, there is wide variation in the number of EI procedures, the type of EI experiences, and the nature of organized training for this procedure in PCCM programs across the USA [7, 8]. In one survey of PCCM program directors (PDs), 14% of programs reported providing no bedside ICU intubation experiences and 5% reported no formal EI training methodology at all [8]. A separate national survey of PCCM PDs and fellows documented that as many as 67% of programs had no protocol for teaching EI and also noted significant discrepancy between PD and fellow perceptions of training for EI [9]. Forty percent of PCCM trainees felt they would *not* be proficient in EI upon completion of training [9].

On average, PCCM PDs felt that trainees required 33 EI experiences to become proficient in this procedure [9]. A similar study found that 2/3rds of PCCM PDs felt that < 39 direct laryngoscopy experiences were sufficient to obtain competence [8]. In that same study, 67% of PDs reported that their fellows performed less than 50 intubations total during their training [8]. However, a recent systematic review of 19,108 intubations performed by anesthesia residents and students concluded that many more than 50 experiences are likely required to achieve competence in *non-elective* EI [10]. Similarly, a large single-center review of pediatric critical care trainees revealed that at least 50 endotracheal intubations are required to attain a 90% overall success rate in out-of-operating-room intubation [11]. One small study concluded that at least 75 procedures are required for emergency medicine trainees to achieve competence in emergent EI [12]. A recent analysis of close to 1000 ICU intubations performed predominantly by PCCM providers revealed a significant increase in the lowest oxygen saturation experienced by critically ill adults undergoing tracheal intubation between 100 and 200 previous operator EIs [13]. Still others advocate for 200 intubations to achieve independent practice in EI in the ICU [14]. Finally, a recent study concluded that greater than 240 experiences are required for competence in EI during cardiopulmonary resuscitation [15]. Satisfactory training for EI in the critically ill likely requires a high number of procedures to achieve competence.

Many programs utilize airway rotations with operating room (OR) experiences for EI training [8]. However, compared to those performed in the OR, EIs performed in the ICU are associated with challenging glottic visualization, higher incidence of “difficult” airways, increased the need for adjunct devices, lower first-pass success, higher incidence of complications, and higher failure rates [6]. Not surprisingly, trainee learning curves vary across environments, with competence in elective OR intubation reported after as few as 43 experiences, but far greater for non-elective procedures [10–14, 16]. While OR EI experiences contribute to attainment of competence in this procedure, they may not offer sufficient situational, physiologic, or anatomic complexity to obviate the need for ICU EI experiences.

Additionally, most programs utilize airway simulators for EI training [8]. However, modern airway management simulators have airway dimensions and tissue compressibility characteristics that significantly differ from those of humans [17, 18]. Similarly, individual manikins differ such that competence achieved on one model may not translate to competence in others [19]. Overall however, a recent systematic analysis of 17 studies concluded that simulation-based airway management training is no better than non-simulation based training [20]. Current recommendations support airway management simulation as an adjunctive tool to bridge the gap between classroom instruction and practical application [18, 21] (Table 1).

**Table 1:** Immediate recommendations

Immediate recommendations to reduce variation and foster competence	
Continued multispecialty research to establish criteria for competence in airway management	
Shift from number-based assessment toward individualized longitudinal competence-based assessments	
Adoption of modern, evidence-based procedural training methodologies (e.g., mastery learning, video laryngoscopy with real-time coaching, expert modeling)	
Increased reliance on frequent real-time MICU patient airway management experiences	
Simulation and operating room experiences as adjuncts to out-of-operating-room patient airway management experiences	
Increased training collaboration between PCCM specialists and anesthesiology, emergency medicine, and pediatric intensivist intubation training experts	
Establishment of a national multispecialty and PCCM-specific working groups to draft guidelines for training in airway management	

Importantly, there is increasing recognition that experience alone may not be efficient or effective for attainment of procedural competence [22]. At the same time, increasing the use of high flow nasal cannula and non-invasive positive pressure ventilation, non-PCCM providers managing the airway in the ICU, and increasing number of trainees competing for EIs may be resulting in fewer EI experience for individual trainees. Thankfully, recent PCCM studies have shown that improvements in EI education *do* translate to improved first pass success, decreased incidence of hypoxia, and decreased incidence of tube misplacement [23–25]. That is, several interventions to improve the *quality* (rather than quantity) of intubation experiences have shown promise for accelerating attainment of trainee competence and improving patient outcomes. Most notably, deliberate practice—intentional sequential experiences with expert observation and immediate feedback for the deliberate goal of improvement—has been shown to improve learner and patient outcomes in central venous catheterization, lumbar puncture, pediatric resuscitation, paracentesis, hernia repair, and cricothyroidotomy, as well as endotracheal intubation [26–29]. Similarly, expert “coaching”—structured, real-time, expert feedback—has been shown to optimize the quality of EI training encounters and improve neonatal intubation success rates [30]. Likewise, expert modeling—observation of expert demonstration of expected goal behaviors and performance—has been shown to improve behavior and technical skills in neonatal resuscitation and has been proposed as a means to accelerate attainment of competence in EI [31, 32]. Real-time video as well as delayed audio and video recording have also been shown to facilitate deliberate practice, expert coaching, and expert modeling interventions [29, 32, 33]. Incorporation of procedural training advancements will enhance the educational value of intubation experiences and accelerate acquisition of skills.

It has been argued that the anesthesiologist should be the specialist of choice for airway management in the ICU [3]. PCCM specialists have argued the opposite, while emergency medicine specialists have sought to show equivalent outcomes between proceduralists of differing specialties [34–36]. Importantly, the harm caused by medical “silos” is well recognized, as is the benefit of intra-specialty collaboration [37–40]. Realistically, it is training and experience, rather than specialty, that most determines proficiency in this procedure [41]. As such, PCCM trainees would benefit from deliberate program efforts to ensure access to non-elective and ICU EI experiences. Likewise, both trainees and patients would benefit from EI training that leverages the perspectives, expertise, and research of all invested specialties even across borders where feasible.

Finally, while training guidelines have been generated for many medical procedures common to the ICU, and guidelines have been generated for EI training in other environments, no consensus guidelines have been established for training in this procedure in this environment [42–45]. Similarly, while competency assessment tools have been established to facilitate modern bronchoscopy training, few such tools exist for training in EI [46]. Such guidelines and tools would likely do much to decrease variation and increase trainee competence**.**

## Conclusions

In summary, the current PCCM EI training environment is characterized by wide variation in practice, and graduating intensivists who consider themselves unprepared for this procedure [8, 9]. Overall, we recommend deliberate, longitudinal, individualized, competency-based airway management programs characterized by simulated and OR practice *as a bridge* to high-frequency real-life ICU EI experiences (Table 1). Incorporation of modern procedural training advances (i.e., mastery learning/deliberate practice techniques, expert modeling, video laryngoscopy with real-time coaching) will be imperative to improve the educational value of individual training experiences and to accelerate acquisition of proficiency. Even with modern, individualized, competency-based training, however, a high volume of EI experiences in the ICU will be required for trainee proficiency in that setting. Deliberate effort to provide trainees with sufficient non-elective EI experiences will likely be required. Excellence in this endeavor will require not only the incorporation of knowledge accumulated by all invested specialties, but also active multispecialty training collaboration. Finally, consensus PCCM EI training guidelines and evidence-based assessment tools will be crucial to decrease variation and ensure standardized trainee competence.

## Data Availability

N/A

## Abbreviations

EI: Endotracheal intubation
ICU: Intensive care unit
PCCM: Pulmonary and critical care medicine
PD: Program director
OR: Operating room
